# Study on Patient Satisfaction in the Government Allopathic Health Facilities of Lucknow District, India

**DOI:** 10.4103/0970-0218.45372

**Published:** 2009-01

**Authors:** Ranjeeta Kumari, MZ Idris, Vidya Bhushan, Anish Khanna, Monika Agarwal, SK Singh

**Affiliations:** Department of Community Medicine, King George Medical University, Lucknow, Uttar Pradesh, India

**Keywords:** Patient satisfaction, health facilities, India

## Abstract

**Background:**

The outcome of any disease is influenced by the decisions to seek care, timely arrival at appropriate diagnostic and treatment services and the receipt of adequate care from service providers. Satisfaction in service provision is increasingly being used as a measure of health system performance. Satisfaction manifests itself in the distribution, access and utilization of health services. Objectives: To determine the areas and causes of low satisfaction among the patients and suggest methods for improvement.

**Materials and Methods:**

Multistage stratified random sampling was used to select the government allopathic health facilities of Lucknow district and systematic random sampling for the selection of the patients for the interview.

**Results:**

The accessibility was difficult in 42% patients and waiting time more than 30 min for 62.5% of those attending the tertiary level health facility. The satisfaction with the duration of the outpatient department (OPD) (64.6%) and the presence of signboards (46.6%) was also found to be low. The overall satisfaction regarding the doctor-patient communication was more than 60% at all the levels of health care facilities but that with the examination and consultation was less than 60% at the primary level as compared to more than 80% elsewhere. The most important motivating factor for the visit to the tertiary (48.2%) and secondary level (71.9%, 67.1%) of health facilities was the faith on doctors or health facility.

**Conclusions:**

The level of patient satisfaction is severely deficient in several areas and needs improvement for the achievement of optimal health of the people.

## Introduction

The quality of service in health means an inexpensive type of service with minimum side effects that can cure or relieve the health problems of the patients.(1) It is easier to evaluate the patient's satisfaction towards the service than evaluate the quality of medical services that they receive.(2) Therefore, a research on patient satisfaction can be an important tool to improve the quality of services.(3,4)

Other industries have been paying attention to customer satisfaction for years. “Health care is the only industry - service or manufacturing - that for years has left the customer out of it. This is an absolutely prehistoric thinking. To ignore the input from the patient, to ignore the customer, to say the customer's desires are irrelevant is not living with reality”.(3)

Health care consumers today, are more sophisticated than in the past and now demand increasingly more accurate and valid evidence of health plan quality. Patient-centered outcomes have taken center stage as the primary means of measuring the effectiveness of health care delivery. It is commonly acknowledged that patients’ reports of their satisfaction with the quality of care and services, are as important as many clinical health measures. Health care organizations are operating in an extremely competitive environment, and patient satisfaction has become a key to gaining and maintaining market share.

Patient satisfaction with the healthcare services largely determines their compliance with the treatment and thus contributes to the positive influence on health. This study was therefore undertaken with the aim to find out the level of patient satisfaction related to different parameters of quality health care including the prescription at public health facilities in the Lucknow district, a centrally placed, capital city of the most populous Indian state - Uttar Pradesh.(5) Being the capital city of Uttar Pradesh, it leads in the provision of health care to its people. It has various health care facilities providing different levels of health care. While it has Sanjay Gandhi Post Graduate Institute for providing superspeciality care, it has the renowned King George Medical University with its state-of-art Trauma centre for the tertiary level health care. The health system is also supported by the district hospital (DH), community health centers (CHC) and the primary health centers (PHC) for the secondary and primary level of care, respectively.

## Materials and Methods

Study design: The study design was cross-sectional.

Study population: The present study was conducted among the patients attending the outpatient department (OPD) of government health facilities of Lucknow district.

Period of study: The period of survey was from May 2006 to August 2006.

### Sampling

Sampling frame: The sampling frame consisted of all the allopathic public health facilities of Lucknow district at the tertiary level, secondary level, and the primary level. The “public health facilities” in the present study implies all the Government health facilities.

Sample size: The sample size was calculated using the formula, *n* = *Z*^2^_(1-α/2)_*pq*/*d*^2^ (where *Z*_(1-α/2)_ = 1.96 at 95% confidence; *p* = prevalence of patient satisfaction, *q* = 1-*p*; *d* = absolute allowable error. For this study, we presumed maximum variability, hence *p* = 0.5; *q* = 0.5; *d* =5%. Sample size thus yielded was of 384. Adding a 10% for incomplete answers, the total number came out to be 422. As the interview was to be taken at four types of public health facilities i.e., Medical College (MC), DH, CHC and PHC, the calculated sample size was multiplied by 4 to obtain the sample size of 1688. The data was analyzed for 1625 patients only who had provided the complete answers.

Sampling technique: Multi-stage stratified random sampling technique was used to select representative patients attending the public health facilities of Lucknow district. At first stage i.e., at the tertiary level, MC was selected. At the second stage i.e., at the secondary level, Balrampur DH and two CHCs were randomly selected. At third stage i.e., at the primary level, two PHCs each under the two selected CHCs were randomly selected.

Further at the MC, the sampling population (844) was interviewed from the 10 most frequented OPDs (Medicine, General surgery, Obstetrics and Gynecology, Paediatrics, Orthopedics, Otorhinolaryngology, Ophthalmology, Cardiology, Neurology, Tuberculosis and Chest diseases) according to probability proportion to size based on the past year's OPD attendance.

At the DH, a total of 422 patients with 105 patients from each of the OPDs of Medicine, General surgery, Obstetrics and Gynecology and Paediatrics were interviewed. Similarly, 105 patients were interviewed from each of the two randomly selected CHCs and 53 new patients from each of the four PHCs, respectively.

Inclusion criteria: A “new” or “referred” patient attending the OPD of the respective health care facility.

Exclusion criteria: Patients working in the health care facility and follow-up patients attending the OPD of the respective health care facility were excluded from the study.

Selection of patient: The patients attending the OPD of the respective health care facility were selected for the interview by systematic random sampling. Depending upon the previous attendance of the particular department and the time taken to complete the interview, a random number was chosen and every *n*^th^ patient was selected for the interview. This process was continued till the required sample size was completed.

Tools of data collection: Permission to conduct the study was taken from the superintendents of the concerned health care facility. All the patients were interviewed after they had consulted the doctor. Informed verbal consent was taken from all the participating patients before the start of the interview after telling them about the objective of the study and the approximate time that will be involved in the completion of the interview. The prescribing doctor was largely kept unaware of the procedure, except in unavoidable circumstances, to avoid the bias in their behavior with the patient.

A quantitative structured interview schedule (Annexure I) was used to record information taking the key elements of sociodemographic characteristics of the patients attending the outpatient health care facility, patient satisfaction regarding accessibility of health services, waiting area and waiting time, examination room and clinical consultation and the drug prescription. Adding the numerators of all the variables taken into consideration and dividing it by the sum of all the denominators of the variables calculated the overall satisfaction regarding a particular aspect. The satisfaction was graded as unsatisfactory (0-20%), satisfactory (20-40%), good (40-60%), very good (60-80%) and excellent (80-100%).

### Analysis

Data was tabulated on Microsoft Excel sheet and analysed using the software Epi Info version 6 and Microsoft Excel (Analysis toolpak) for Windows. Discrete data was analysed using Pearson's Chi-square test for normal distribution. *P* values <0.05 were considered significant.

## Results

We observed in the present study that, the primary level health facilities were the most easily accessible (88.3%), affordable (76.1%), required less travel time. About one third of those attending the tertiary health facility were unsatisfied with the duration of the OPD [Table 1].

**Table 1:** Patient satisfaction regarding accessibility of health services in the government health facilities

Level of care accessibility factors	Tertiary		Secondary				Primary		Total (1625)	
					
	Medical college (817)		District hospital (401)		Community health centre (202)		Primary health centre (205)			
					
	*n*	%	*n*	%	*n*	%	*n*	%	*n*	%
Accessibility										
Easy	474	58.0	305	76.0	158	78.2	181	88.3	1118	68.8
*P* value	*0.000			†0.0004			‡0.000			
Mode of transport										
Walking	41	5.0	66	16.4	66	32.7	143	69.7	316	19.4
Private automated transport	95	11.6	25	6.2	15	7.4	6	3.0	141	8.6
Private non-automated	39	4.8	15	3.7	38	18.8	34	16.6	126	7.7
Public automated transport	572	70.0	204	48.9	82	40.6	22	10.7	880	54.2
Public non-automated	70	8.6	91	22.7	1	0.5	0	0	162	9.9
*P* value				<0.0001						
Parking space§										
Available	133/*134*	99.2	34/*34*	100	48/*48*	100	42/*42*	100	257/*258*	99.6
*P* value	*1.000			‡1.000						
<30 min	206	25.2	129	32.2	113	56	134	65.4	582	35.8
30 -60 min	206	25.2	203	50.6	75	37.1	49	23.9	533	32.8
>60 min	405	49.6	69	17.2	14	6.9	22	10.7	510	31.4
*P* value				< 0.0001						
Cost for reaching										
Affordable	577	70.6	258	64.3	137	67.8	156	76.1	1128	69.4
*P* value	*0.04			†0.004			‡0.119			
Timing of OPD										
Convenient	750	91.8	344	85.8	170	84.1	177	87.6	1441	88.7
*P* value	*0.000			†0.69			‡0.01			
Other convenient time										
Afternoon	16	1.9	5	1.2	1	0.5	2	0.9	24	1.5
Evening	195	23.8	118	29.4	81	40.1	35	17.1	429	26
*P* value				< 0.15						
Duration of OPD										
Sufficient	528	64.6	357	89.0	197	97.5	205	100	1287	79.2
*P* value	*0.000			†0.000			‡0.000			

*P*-values <0.05 are significant

* Association of variables between tertiary and secondary level

† Association of variables between secondary and primary level

‡ Association of variables between tertiary and primary level

§ Numbers in italics are the denominator for that variable

Enquiries about the waiting area and waiting time in the public health facilities revealed a significantly high satisfaction with respect to the presence of signboards (100%), waiting time of less than 30 minutes (99.5%) and overcrowding (32.8%) in the patients attending the primary level health facilities, while that regarding the availability (2.9%) and cleanliness of the toilets (2.9%) was miserable. On the other hand, the satisfaction regarding the waiting area (92.9%), availability of seats (81.4%), availability (44.7%) and cleanliness of the toilets (31.3%) was highest at the tertiary level health facilities [Table 2].

**Table 2:** Patient satisfaction regarding waiting area and waiting time in the government health facilities

Level of care variables	Tertiary		Secondary				Primary		Total (1625)	
					
	Medical college (817)		District hospital (401)		Community health centre (202)		Primary health centre (205)			
					
	*n*	%	*n*	%	*n*	%	*n*	%	*n*	%
Signboard to guide										
Present	381	46.6	286	71.3	25	12.4	205	100	897	55.2
Already know	160	19.6	36	8.9	108	53.4	0	0	304	18.7
*P* value	*0.0009			†0.000			‡0.000			
Waiting time										
<30 min	306	37.4	162	40.4	133	65.8	204	99.5	805	49.5
Satisfied	304	99.3	158	97.5	100	75.2	202	99.0	764	94.9
>30 min	511	62.5	239	59.6	69	34.2	1	0.5	820	50.4
Satisfied	146	28.6	24	10.0	11	15.9	0	0	181	22.0
Waiting area										
Clean	759	92.9	357	89.0	169	83.6	141	68.8	1426	87.7
*P* value	*0.0003			†0.000			‡0.000			
Seats available										
Enough	665	81.4	185	46.1	77	38.1	118	57.6	1045	64.3
*P* value	*0.000			†0.0004			‡0.000			
Overcrowding	471	57.6	313	78.1	176	87.1	67	32.8	1027	63.2
present										
*P* value	*0.000			†0.000			‡0.000			
Drinking water available										
Yes	305	37.3	267	66.7	95	47.0	74	36.1	741	45.7
No	206	25.2	40	9.9	70	34.7	104	50.7	420	25.9
Don’t know	306	37.5	94	23.4	37	18.3	27	13.2	464	28.5
*P* value	*0.000			†0.000			‡0.000			
Toilets available										
Yes	365	44.7	166	41.4	71	35.1	6	2.9	608	37.4
No	116	14.2	58	14.5	71	35.1	129	62.9	374	23
Don’t know	336	41.1	177	44.1	60	29.8	70	34.2	643	39.6
*P* value	*0.0004			†0.000			‡0.000			
Toilets										
Clean	256	31.3	134	33.4	47	23.3	6	2.9	443	27.3
*P* value	*0.594			†0.000			‡0.000			

*P*-values <0.05 are significant

* Association of variables between tertiary and secondary level

† Association of variables between secondary and primary level

‡ Association of variables between tertiary and primary level

Table 3 shows that the overall patient satisfaction regarding doctor patient communication decreased significantly from tertiary level (73.3%) through secondary (68.0%, 66.1%) to primary level (60.5%) health facilities. The total satisfaction regarding explanation about the disease (54.3%), treatment (57.6%), investigations (59.4%) and advice about prevention (21.6%) was quite low.

**Table 3:** Patient satisfaction regarding doctor patient communication in government health facilities

Level of care variables of doctor-patient communication	Satisfaction									
	
	Tertiary		Secondary				Primary		Total (1625)	
					
	Medical college (817)		District hospital (401)		Community health centre (202)		Primary health centre (205)			
					
	*n*	%	*n*	%	*n*	%	*n*	%	*n*	%
Listening of complaints	778	95.2	374	93.3	186	92.1	189	92.2	1527	93.9
Explanation about										
Disease	509	62.3	207	51.6	101	50	65	31.7	882	54.3
Treatment	579	70.9	206	51.4	88	43.6	63	30.7	936	57.6
Investigations discussed*	137/*261*	52.5	66/*83*	79.5	20/*31*	64.5	4/*7*	57.1	227/*382*	59.4
Advice about prevention	229	28.0	72	17.9	29	14.3	22	10.7	352	21.6
Behaviour										
Doctor	797	97.5	394	98.2	199	98.5	201	98.0	1591	97.9
Para/non medical staff	756	92.5	375	93.5	199	98.5	204	99.5	1534	94.4
Overall satisfaction (%)	73.3		68.0		66.1		60.5		69.6	
*P* value	†0.000			‡0.000			§0.000			

*P*-values <0.05 are significant

* Numbers in italics are the denominator for that variable

† Association of variables between tertiary and secondary level

‡ Association of variables between secondary and primary level

§ Association of variables between tertiary and primary level

The overall satisfaction regarding examination and consultation were significantly higher at the tertiary (81.6%) and secondary (81.3%) level, as compared to the primary level health facilities (59.6%). Absence of a separate place for examination at the primary level resulted in high dissatisfaction [Table 4].

**Table 4:** Patient satisfaction regarding examination and consultation in government health facilities

Level of care variables examination and consultation	Satisfaction									
	
	Tertiary		Secondary				Primary		Total (1625)	
					
	Medical college (817)		District hospital (401)		Community health centre (202)		Primary health centre (205)			
					
	*n*	%	*n*	%	*n*	%	*n*	%	*n*	%
Consult the main doctor										
Yes	292	35.7	196	48.9	17	8.4	24	11.7	529	32.5
Able to	168	57.3	169	86.2	14	82.3	4	16.6	355	67.1
>1 patient in the room										
Present	570	69.7	262	65.3	124	61.3	111	5.4	1067	65.6
Comfortable	418	73.3	190	72.5	99	79.8	104	93.7	811	76.0
Separate place for examination										
Present	566	69.3	182	45.4	75	37.1	0	0	823	50.6
Cleanliness	441	77.9	127	69.7	51	68.0	0	0	619	75.2
Examination	711	87.0	318	38.9	153	75.7	109	53.2	1291	79.4
All patients treated equally	631/*693*	91.0	304/*309*	98.4	138/*140*	98.5	149/*152*	98.0	1222/*1294*	94.4
Total time of consultation										
(Mean ± SD) in min	6.6±3.7		5.4± 2.4		5.3±2.6		2.8±1.5		5.7±3.3	
Satisfied	696	85.2	323	80.5	156	77.2	109	53.2	1284	79.0
Overall satisfaction (%)	81.6		81.7		80.4		59.6		79.0	
*P* value	*0.76			†0.000			‡0.000			

*P*-values <0.05 are significant

* Association of variables between tertiary and secondary level

† Association of variables between secondary and primary level

‡ Association of variables between tertiary and primary level

History taking about the allergy to drugs was equally poor in all the health facilities (0.6%) while that about the use of other drugs (30.3%) as well as information imparted about the side effects (1.9%) and about returning immediately (19.7%) due to the adverse effects of drugs was higher at the MC in comparison to other facilities, but unsatisfactory on the whole. A significantly greater proportion of the patients attending the PHC wanted a change in the form of the drugs (40.9%) as well as expected tonics (45.8%) [Table 5].

**Table 5:** Patient satisfaction regarding the drug prescription in the government health facilities

Level of care variables	Satisfaction										
	
	Tertiary		Secondary				Primary		Total (1541)		*P* value
						
	Medical college (749)		District hospital (387)		Community health centre (200)		Primary health centre (205)				
					
	*n*	%	*n*	%	*n*	%	*n*	%	*n*	%
Asked about											
LMP in females*	112/*373*	30.0	102/*197*	51.7	28/*105*	26.6	10/*95*	10.5	252/*770*	32.7	0.000
Allergy	8	2.1	1	0.2	0	0	1	0.5	10	0.6	0.22
Use of other drugs	224	30.3	36	9.3	18	9	1	0.5	279	18.1	0.00
Overall satisfaction %	18.6		14.3		9.1		2.4		15.1		
Verbal directions given											
Doses	560	75.9	185	47.9	136	68.3	166	80.9	1047	67.9	0.000
Frequency	560	75.9	185	47.9	136	68.3	165	80.5	1046	67.8	0.000
Route	557	75.5	184	47.6	137	68.4	166	80.9	1044	67.7	0.000
Nonpharmacological											
treatment*	82/*120*	68.3	5/*10*	50	1/*5*	20	1/*3*	33.3	89/*138*	64.5	0.06
Overall satisfaction %	75.3		47.8		68.1		80.6		67.7		
Told about											
Side effects	14	1.9	2	0.5	0	0	1	0.5	17	1.1	0.04
Returning*	161/*817*	19.7	66/*401*	16.4	29/*202*	14.3	2/*205*	1.0	258/*1625*	15.8	0.000
immediately											
Overall satisfaction %	11.2		8.6		7.2		0.7		8.7		
Follow up visit											
Told*	646/*817*	79.1	249/ *401*	62.1	138/ *202*	68.3	127/*205*	61.9	1160	71.4	0.000
Satisfied	518	80.2	226	90.7	81	58.7	84	66.1	909	78.3	0.00
Want change in the form of drugs	33	4.5	94	24.3	57	28.6	84	40.9	268	17.5	0.00
Form of the drug											
Syrup	17	51.5	45	47.8	25	43.8	29	34.5	116	43.3	0.001
Injections	7	21.2	46	48.9	27	47.3	39	46.4	119	44.4	
Eye/Ear dps	6	18.2	2	2.1	3	5.2	9	10.7	20	7.5	
Ointment	2	6.1	13	13.8	6	10.5	10	11.9	31	11.5	
Others	2	6.1	2	2.1	0	0	1	1.2	5	1.8	
Expect tonics	76	9.3	164	40.9	85	42.0	94	45.8	419	25.8	0.00

*P*-values <0.05 are significant

* Numbers in italics are the denominator for that variable

Regarding the time spent in seeking medical care as perceived by the patient in various public health facilities, it was observed that the average waiting time for registration at the tertiary care health facility (8.1 ± 9.1 minutes) was highest followed by secondary level health facility and least at the primary level (3.8 ± 2.8 minutes).

It can be observed from [Table 6] that the most important motivating factor for the visit to the tertiary and secondary level of health facilities was the faith on doctors or health facility, followed by the availability of specialists (43%, 63%). On the other hand, the proximity of the health facility to the residence (67.1%), followed by faith on doctors or health facility (55.4%) and cost-effectiveness (34.3%) were more important at the primary level.

**Table 6:** Motivating factors for the visit to the particular health facility in the new patients*

Level of care factors	Tertiary		Secondary				Primary		Total (1469)	
					
	Medical college (682)		District hospital (382)		Community health centre (201)		Primary health centre (204)			
					
	*n*	%	*n*	%	*n*	%	*n*	%	*n*	%
Cost effective	59	8.6	145	37.9	56	27.8	70	34.3	330	22.4
Faith on doctors/health facility	329	48.2	275	71.9	135	67.1	113	55.4	852	58.0
Facilities for investigations/operation present	11	1.6	5	1.3	0	0	0	0	16	1.0
Someone known works in the health facility	55	8.0	8	2.0	2	1.0	2	0.9	67	4.5
Specialists available	282	41.3	241	63.0	16	7.9	2	0.9	541	36.8
No benefit from other facilities	218	31.9	60	15.7	23	11.4	20	9.8	321	21.8
Near to residence	43	6.3	61	15.9	134	66.6	137	67.1	375	25.5
Someone known lives in Lucknow	22	3.2	9	2.3	0	0	0	0	31	2.1
Others	66	9.6	38	9.9	9	4.4	4	1.9	117	7.9

* Includes multiple responses

## Discussion

The present study was an attempt to assess the level of satisfaction of the patients with the various aspects of health care in the allopathic government health facilities of Lucknow district. Very few similar studies have been done and therefore we lack the data for comparison. Yet, the findings of the survey are quite helpful if they are transformed into actions for improving the quality of health care.

Accessibility is one of the principles of Health for All, as stated in Alma Ata declaration on primary health care.(6) Although, the large catchment area of the tertiary health facilities make it less accessible, yet people travelled by the public automated transport for more than an hour to reach there to receive specialized services. Our findings are consistent with those of Gadallah *et al*,(7) with respect to the accessibility and the traveling time. The affordability of the cost involved in reaching the health facility by almost all signifies the readiness of the patients to pay for their health. The demand for evening OPD services can be an important finding regarding the reforms that need to be made for making the health services more user-friendly. The decreased level of satisfaction with the duration of the OPD at the tertiary level could be attributed to a number of factors such as short duration of four hours, compounded by late arrival, relative lack of appropriate signboards and misleading of the ignorant patients by people from private agencies, adding to the cost and suffering.

The registration time and waiting time at the primary level was different to the observation of Dr. Syed Mohamed Aljunid(8) in his study in Malaysia where the patients waited for 52 minutes on an average. Differences in satisfaction with long waiting time as compared to other studies by Dr. Syed Mohamed Aljunid,(8) van Uden *et al*(9) and Mahfouz *et al*,(10) could be attributed to the differences in the perceptions and expectations of the people. Reduction of the waiting time by triage of the patients and sending them to the appropriate doctor would save their time and also provide appropriate treatment. The waiting time and area could also be utilized to provide health education to the people. The unsatisfactory availability of drinking water (45.7%) and toilet facilities (37.4%) as well as the cleanliness of the toilets (27.3%) were similar to those of Srilatha Sivalenka(11) and Peerasak Lerttrakarnnon *et al*,(12) who also found these as the major areas of concern in their study.

The satisfaction regarding the listening of the complaints and the behavior of the doctors and the paramedical staff was similar to that recorded by Peerasak Lerttrakarnnon *et al*,(12) in their study, while it was higher than that reported by Janko Kersnik *et al*,(13) who found it to be 69.1% and 56.9%, respectively. Our findings regarding physical examination corroborate with those of Janko Kersnik *et al*,(13) who observed a satisfaction of 55.3%. The relatively low satisfaction at the primary level health facilities might result in loss of faith and non-compliance with the treatment. The average consultation time was similar to that observed by Desta *et al*,(14) Hazra *et al*(15) and Mallet *et al*,(16) while it was considerably less from that of Pati(17) (7 min) and Guyon *et al*,(18) (54 s).

Serious medication errors could result from inappropriate history taking prior to and inadequate instructions while prescribing the drugs as observed in our study. A proper drug dispensing system may help the patients overcome the dissatisfaction regarding the form and duration of the drugs to prevent noncompliance as well as avoid grave consequences of the medication errors.

Improvement of the skills of doctor-patient communication and other relevant areas would go a long way to enhance the level of satisfaction of the patients, considering the fact that most of the patients were drawn to the health facility because of their faith. The cost effectiveness of the services provided would also go a long way to maintain the bond between the doctors and the patient for the achievement of the optimal level of health of the people.

## Limitations

Due to non-availability of similar studies in India, the sample size was calculated taking the percentage of patient satisfaction as 50%. But, since it provides us with maximum variability, the sample size is considered appropriate. The responses of the patients depend upon their personality and their perceptions. Some may be satisfied with average services while others may be dissatisfied with even the best. The results also need to be compared with the other major group of health providers’ i.e., private practitioners to evaluate the differences in the quality of health care but could not be done due to the paucity of resources.

## Conclusions

An attempt to evaluate the level of patient satisfaction related to different parameters of quality health care at the health facilities has provided us with the certain areas that need corrective efforts to improve hospitals’ service quality. Infrastructure and architectural corrections need to be made to enhance the comfort and satisfaction of the patients. There is a need to channelize the patients through the hierarchical levels of health care to prevent undue burden on the tertiary health facilities. Certain improvements are also needed in the waiting area by making it informative and comfortable. Also, there is an imperative need to communicate effectively with the patients about their disease and the treatment specially the largely ignored and the most efficient preventive aspect to allay their fears, remove misconceptions, comply with the treatment and develop confidence in the health system for achieving the standards of good health.
